# Adjective Metaphors Evoke Negative Meanings

**DOI:** 10.1371/journal.pone.0089008

**Published:** 2014-02-19

**Authors:** Maki Sakamoto, Akira Utsumi

**Affiliations:** Department of Informatics and Engineering, The University of Electro-Communications, Tokyo, Japan; Emory University, United States of America

## Abstract

Previous metaphor studies have paid much attention to nominal metaphors and predicative metaphors, but little attention has been given to adjective metaphors. Although some studies have focused on adjective metaphors, they only examined differences in the acceptability of various types of adjective metaphors. This paper explores the cognitive effects evoked by adjective metaphors. Three psychological experiments revealed that (1) adjective metaphors, especially those modified by color adjectives, tend to evoke negative effect; (2) although the meanings of metaphors are basically affected by the meanings of their vehicles, when a vehicle has a neutral meaning, negative meanings are evoked most frequently for adjective metaphors compared to nominal and predicative metaphors; (3) negative meanings evoked by adjective metaphors are related to poeticness, and poetic metaphors evoke negative meanings more easily than less poetic metaphors. Our research sheds new light on studies of the use of metaphor, which is one of the most basic human cognitive abilities.

## Introduction

The following are expressions from German poetry by Georg Trakl [1]:

1. Immer klangen von dämmernden Türmen die blauen Glocken des Abends. (An einen Frühverstorbenen)

‘The blue evening bells still ring from the twilit towers’

1. Die weiße Stimme sprach zu mir: Töte dich! (Offenbarung und Untergang)

‘The white voice talked to me: Kill yourself!’

1. Schwemut und purpurnes Lachen (Abend in Lans)

‘Gloom and deep red laugh’Trakl uses color adjective metaphors in a very striking way throughout the poem. (1) is an example of adjective metaphors created from *blue* ‘blau’ in German. There are other expressions using *blue* ‘blau’ in his poems; *the blue cry* ‘die blaue Klage,’ *in the blue evening the figure of the dead* ‘im blauen Abend der Toten Gestalt,’ and *A blue animal is scared of death* ‘Ein blaues Tier will sich vorm Tod verneigen.’ These expressions emphasize negative images working in negative contexts (e.g., about death). The last example evokes a metaphorically negative image by describing an animal as blue. (2) is an example of an adjective metaphor created from *white* ‘weiß.’ There are other expressions such as *white sorrow* ‘weiße Traurigkeit’ and *The white offspring’s dark future* ‘Die weißen Enkel dunkle Zukunft.’ These expressions also emphasize negative images working in negative contexts (e.g., about death). (3) is an example of an adjective metaphor created from *deep red* ‘purpurn.’ There are many other examples using *deep* to be found in Trakl’s poems; *deep red plague* ‘purpurne Seuche,’ *The deep red curses* ‘Die purpurnen Flüche,’ *in deep red dreams pain and agony* ‘in purpurne Träume Schmerz und Plage,’ and *the deep red sufferings* ‘die purpurnen Marten.’ These expressions also emphasize negative images.Sakamoto [2] analyzes the meanings of composite expressions of nouns modified by color terms (e.g., red, blue, yellow, black, and white) collected from a Japanese corpus containing literary texts. She reports that there are many examples of color metaphors emphasizing negative images, as shown in the following examples.

1. aoi kannen-teki-na keno-kan noho-ga tsuyo katta.

‘I had a rather blue and ideological disgusted feeling.’

1. kentai-no aoi uta

‘weary blue song’

1. nigotta, shiroi yokujou

‘dark, white sexual desire’The expressions given above seem to evoke negative images, but in this paper we test this assumption through psychological experiments. Furthermore, although the above are examples of color adjective metaphors, we will examine not only color adjective metaphors but also other types of adjective metaphors more thoroughly to investigate whether evoking negative images is a unique feature of adjective metaphors through a comparison with nominal metaphors and predicative metaphors. We also address the issue of why these metaphors evoke negative images.Metaphors have traditionally been thought simply be devices of the poetic imagination that represent a rhetorical flourish―a matter of extraordinary rather than ordinary language. Metaphors have typically been viewed as characteristic of language alone, a matter of words rather than thought or action. Lakoff and Johnson [3], however, have found that metaphors are pervasive in everyday life, not just in language but in thought and action as well. Since the time of those studies, an increasing number of studies have revealed that our conceptual system is largely metaphorical, and that the way we think about what we experience, and what we do every day, is very much a matter of metaphor [4]–[9]. Moreover, controversies have also arisen as this field of research has matured [10]–[12].The prevalence of metaphor in language and thought has motivated a considerable number of cognitive studies on metaphor, particularly on the cognitive mechanisms of metaphor comprehension. Most such studies have focused on nominal metaphors such as “*My job is a jail*” [13]–[18] and predicative metaphors such as “He *shot down* all of my arguments” [3], [19]. In this study, however, we focus on adjective metaphors, which are metaphors in which adjectives serve as the vehicle to modify nouns as the topic of the metaphor. (7)–(9) are examples of adjective metaphors.

1. The old woman had an open heart.
2. The rich man had a cold heart.
3. The stone statue had a cold smell.

**Figure 1:**
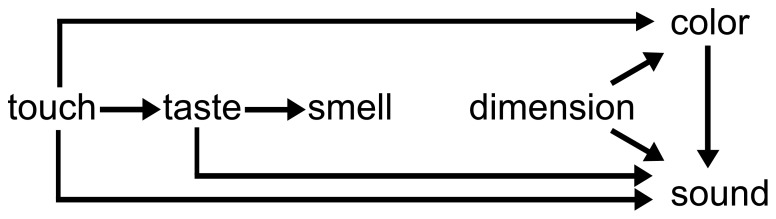
Directionality of synesthetic metaphors proposed by Williams [25]. According to Ullmann’s [24] theory of directionality, a metaphor with a source domain lower in the hierarchy of sense modalities than the target domain should tend to be cognitively more accessible than a metaphor with the reverse direction of domains. Figure 1 shows this directionality, as proposed by Williams [25].

Adjective metaphors are sometimes called synesthetic metaphors. According to prominent theories of metaphor [3]–[5], [9], [11], [20]–[22], all metaphors result from a mapping of some concept from a source domain onto a concept of some target domain as shown in Figure 1. In the case of synesthetic metaphors, the source domain is restricted to concepts of perception, which make up the perceptual domain. The classification of the perceptual domain can be made with the five senses: vision, sound, touch, smell, and taste. Werning, Fleischhauer, andBeşeoğlu [23] call linguistic expressions such as those shown in (8) and (9) synesthetic metaphors, while example (7) would not be considered a synesthetic metaphor because the modifier does not come from a perceptual domain. As shown in examples (7)–(9), synesthetic metaphors comprise a kind of adjective metaphor, in which an adjective denoting the perception of some sense modality modifies a noun’s modality.

Many studies focusing on synesthetic metaphors have examined how the acceptability of synesthetic metaphors can be explained by the pairing of the modalities of adjective modifiers and head nouns. Ullmann [24] discovered “hierarchical distribution,” the theory that synesthetic transfers tend to go from the “lower” to the “higher” sensory modes, namely, touch → taste → smell → sound → sight. Ullmann claims that the qualities of the lower senses should preferentially occur in the source domain, while the qualities of the higher senses should be preferred in the target domain. His theory of directionality thus asserts that a metaphor with a source domain lower in the hierarchy of sense modalities than the target domain should be cognitively more accessible than a metaphor with the reverse direction of domains. Williams [25] examined synesthetic adjectives in conversational English (along with some evidence from other Indo-European languages and Japanese) and developed a more differentiated account of directionality (see Figure 1).

Twenty years after Williams’ study was published, Day [26] compared German and English synesthetic metaphors, and the results suggested that, at least for Germanic languages, and possibly for a fair share of Indo-European languages, the English/German model of synesthetic metaphor ranking holds cross-linguistically. Conversely, Yu [27] has highlighted some cross-linguistic differences in his separate account of directionality with respect to different languages (i.e., a comparison of English and Chinese). Recently, Werning, Fleischhauer, and Beşeoğlu [23] explored factors that enhance the cognitive accessibility of synesthetic metaphors in German.

Other studies on adjective metaphors have investigated whether adjective metaphors are motivated by the perceived resemblance between the two perceptual domains or by correlations in experience. In cognitive linguistics research [3], [28], it has been widely argued that there are two distinct classes of metaphors, namely metaphors based on resemblance and metaphors based on correlation in experience. A metaphor like “Achilles is a lion” is an example of a resemblance-based metaphor. We perceive something in common between stereotypical lions and brave people. Conceptual metaphors like HAPPY IS UP, as in “She is in *high* spirits,” are grounded in correlations based on our real world experiences. The HAPPY IS UP metaphor is grounded in the experience that persons in a positive emotional state have an erect posture, that is, they carry themselves in a positive, tall manner. As for synesthetic metaphors, Taylor [29] argues that synesthetic metaphors (adjective metaphors) are based only on a perceived resemblance or similarity and thus cannot be reduced to experiential correlations. However, Sakamoto and Utsumi [30] argue that certain adjective metaphors are indeed based on correlations. For example, the metaphor “sweet smell” (“amai nioi” in Japanese) seems to be based on correlations in experience. “Sweet smell” is the smell you experience when eating something sweet.

Very few studies, however, have attempted to explore the cognitive effects evoked by adjective metaphors. Therefore, this paper explores such cognitive effects, and in particular synesthetic metaphors as a subcategory of adjective metaphors.

Metaphor comprehension has been recognized by interaction theorists as the process of finding relevant features that constitute the metaphorical meaning from the interaction between a source concept and a target concept [20], [31]–[33]. In this paper we will refer to semantic changes resulting from the interaction between two concept domains as ‘cognitive effects.’ We will see how semantic interactions between the vehicle and topic of adjective metaphors function to shift the meanings of words to the positive pole or the negative pole.

The rest of this article is organized as follows. Section of Experiment 1 presents the first experiment (Experiment 1) conducted to examine the tendencies of cognitive effects associated with adjective metaphors, and whether adjective metaphors, especially those modified by color adjectives, tend to evoke negative images. Based on the results of Experiment 1, Section of Experiment 2 presents our main experiment (Experiment 2) conducted to examine whether the evoking of negative meanings is a unique feature of adjective metaphors through a comparison with nominal and predicative metaphors. Based on the results of Experiments 1 and 2, we explore the possible reasons why adjective metaphors were found to evoke negative meaning. The results of Experiment 3 suggest that negative meanings evoked by adjective metaphors are related to poeticness, and therefore that poetic metaphors evoke negative meanings more easily than less poetic metaphors. The last section discusses the significance and implications of this study and possible directions of future work.

## Methods

### Ethics Statement

All participants in Experiments 2 and 3 provided written informed consent prior to the experiments. Documents about the experimental procedures and written informed consents were presented to an internal organization of the University of Electro-Communications, Tokyo, Japan. Participants in Experiment 1 were recruited through Macromill, Inc., Tokyo, Japan, an organization that maintains a panel of more than 533,579 individuals who have provided informed consent and agreed to participate in web-based online survey research. Participants’ rights are protected based on the company’s privacy policy (http://www.macromill.com/global/privacy/index.html). In total, 3,267 Japanese males and females, aged 20–75, agreed to participate in our experiment.

### Experiment 1

We conducted Experiment 1 to examine the tendencies of the cognitive effects associated with adjective metaphors and to analyze whether the semantic interaction between the vehicle and the topic of a metaphor causes changes to either its negative semantic poles or positive semantic poles.

Because previous studies [34], [35] point out that metaphor comprehension is more likely to be affected by the metaphor vehicle rather than the topic, we initially predicted the semantic changes shown in Table 1. Our predictions basically state that the semantic value of the topic shifts toward the semantic value of the vehicle.

**Table 1:** Predictions of semantic change.

Semantic value	Predicted semantic change
T = V	no change (0)
T<V	change to +
T>V	change to −

**Figure 2:**
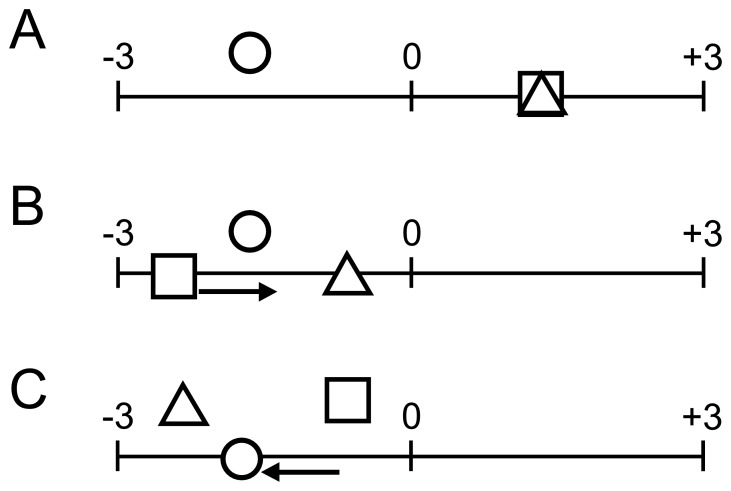
Predictions of semantic change. A triangle indicates a vehicle, a square indicates a topic, and a circle indicates a metaphor. (A) example of no semantic change (0); (B) example of a semantic change toward the positive pole; (C)example of a semantic change toward the negative pole.

The first column in Table 1 shows the classification of potential metaphors based on the value of topics (T) and vehicles (V) in the antagonistic (negative or positive) poles. The second column shows the predicted semantic changes. If the values of topic and vehicle are the same, then their semantic interactions within adjective metaphors should evoke no semantic change. If the value of the topic is smaller than that of the vehicle, however, then their semantic interactions within adjective metaphors should evoke semantic change toward the positive pole. However, if the value of the topic is larger than that of the vehicle, then their semantic interactions within adjective metaphors should evoke semantic change toward the negative pole. Figure 2 A shows an example of no semantic change (0), Figure 2 B shows an example of a semantic change toward the positive pole, and Figure 2 C shows an example of a semantic change toward the negative pole.

We conducted a pre-experiment to choose the materials to be used in the experiment. Thirty Japanese males and females, aged 21–25, participated in the pre-experiment. Materials used for the pre-experiment were 250 Japanese adjective metaphors. These metaphors were made by combining 25 Japanese adjectives denoting perceptions from the five sense modalities with 11 Japanese nouns; *color* (‘iro’ in Japanese), *touch* (‘tezawari’), *voice* (‘koe’), *taste* (‘aji’), *smell* (‘nioi’), *feeling* (‘kimochi’), *dream* (‘yume’), *uneasiness* (‘fuan’), *greed* (‘yokubou’), *affection* (‘aijou’), and *manner* (‘taido’). Twenty-five expressions made by combining adjectives and nouns belonging to the same perceptual domains were excluded as non-metaphorical expressions. These 25 Japanese expressions denoting perceptions from the five sense modalities are shown in Table 2.

**Table 2:** List of adjectives used in Experiment 1.

Color	Touch	Sound	Taste	Smell
*yellow* ‘kiiroi’	*light* ‘karui’	*noisy(1)* ‘urusai’	*tasty* ‘oishii’	*sweetsmelling* ‘kaguwashii’
*blue* ‘aoi’	*hard* ‘katai’	*noisy(2)* ‘yakamashii’	*sweet* ‘amai’	*stinking(1)* ‘kinakusai’
*red* ‘akai’	*soft* ‘yawarakai’	*noisy(3)* ‘sawagashii’	*bitter* ‘shibui’	*stinking(2)* ‘kusai’
*black* ‘kuroi’	*hot* ‘atsui’	*quiet* ‘shizukana’	*hot* ‘karai’	*smelly* ‘namagusai’
*white* ‘shiroi’	*cold* ‘tsumetai’	*loud* ‘kandakai’	*sour* ‘suppai’	*fragrant* ‘koubashii’

Participants were asked to evaluate how easily they understood each metaphor. The ratings were made on a seven-point scale ranging from −3 (very difficult) through 0 (not sure) to +3 (very easy). They were also asked to evaluate how conventional they felt each metaphor to be. The ratings were made on a seven-point scale from −3 (not at all conventional) through 0 (not sure) to +3 (highly conventional). We selected metaphors with a mean value within the range of −2.0 to +2.0 in both scales. This procedure reduced the possibility that differences in the cognitive effects evoked by the adjective metaphors would result from differences in the accessibility or conventionality among the materials used in the experiment. As a result, 158 Japanese adjective metaphors were chosen as the materials to be used in the experiment.

The 3,267 participants were classified into 10 groups, and 18–20 linguistic expressions were assigned to each group. The linguistic expressions assigned to one group were randomly assigned to each participant in that group (e.g., linguistic expressions assigned to Group 1 were randomly assigned to each participant belonging to Group 1).

The participants of Groups 1 and 2 were each assigned 18 adjectives and nouns, and the remaining 8 groups were assigned 19 or 20 metaphorical expressions per participant. They were asked to rate the assigned expressions against the following 15 Semantic Differential (hereafter, SD) scales: *dislike – like, uncomfortable – comfortable, not interesting – interesting, not appropriate – appropriate, dull – sharp, weak – powerful, heavy – light, coarse – delicate, ugly – beautiful, dark – light, unclear – clear, scary – safe, sad – glad, old – new,* and *not salient – salient*. The 15 SD scales were selected from the high factor loading scales among the 34 SD scales used in Kusumi [36] to measure the meanings of the metaphors. The participants were given no instructions with respect to what the scales were measuring. The ratings were made on a seven-point scale ranging from −3 through 0 to +3. We regarded the value −3 as the negative semantic pole and the value +3 as the positive semantic pole.

We classified all of the mean values of the vehicles and topics rated on the 15 SD scales into T = V, T<V, and T>V, considering the prediction given in Table 1. Using a t-test (two-tailed, alpha level of. 05), we regarded the cases that had no significant difference between the mean value of T and V as T = V. The other codes, such as T<V and T>V, describe the cases for which significant differences were found between the mean values of T and V. The total number falling under each classification is given as ‘sum’ in the far right column of Table 3.

**Table 3:** Comparison between predicted semantic changes and actual semantic changes.

Semantic intensity	Predictedchange	Actual change			Sum
		0	+	−	
T = V	0	331	17	261	609
T<V	+	366	230	76	672
T>V	−	119	9	961	1089
Sum		816	256	1298	2370

Unit = cases of SD scales.

To compare the actual semantic changes resulting from our experiment with the predicted semantic changes, we classified the resulting actual semantic change as shown in Table 3. We conducted a t-test (two-tailed, alpha level of. 05), and regarded cases that had no significant difference between the mean values of T and metaphor as ‘no change’ (0), and those cases which had significant differences as changes either to the negative pole (-) or to the positive pole (+). Table 3 shows a comparison between the predicted semantic changes and the actual semantic changes observed in our experiment.

As for the cases which were predicted as no change (0), the proportion of cases showing the same actual change as the predicted change was significantly higher than that showing a change different from the predicted change, χ^2^ (1, N = 609) = 4.612, *p*<.05. Among the cases that showed a change different from the predicted change, the proportion of cases showing the change to the negative pole (-) was significantly higher than the proportion showing the change to the positive pole (+), χ^2^ (1, N = 278) = 214.158, *p*<.001.

As for cases that were predicted to change to the positive pole (+), the proportion of cases showing the same change as the predicted change was lower than that showing a different change, χ^2^ (1, N = 672) = 66.881, *p*<.001. This finding suggests that actual semantic changes do not follow the predicted changes toward the positive pole (+).

As for cases that were predicted to change to the negative pole (−), the proportion of cases showing the same change as the predicted change was significantly higher than that showing a different change, χ^2^ (1, N = 1089) = 637.180, *p*<.001. This result suggests that the actual semantic changes do follow the predicted change toward the negative pole (−).

We also classified the full dataset of 2,370 cases into five types of adjective metaphors, as shown in Table 4. For ‘touch,’ ‘sound,’ and ‘smell’ adjective metaphors that were predicted as having no change (0), the proportion of cases showing the predicted change was significantly higher than that showing a change different from the predicted change, χ^2^ (1, N = 106) = 15.094, *p*<.01 for touch; χ^2^ (1, N = 87) = 21.252, *p*<.001 for sound; χ^2^ (1, N = 127) = 8.574, *p*<.001 for smell. As for ‘taste’ adjective metaphors, there was no significant difference found, χ^2^ (1, N = 130) = 0.769, *p* = .380. On the other hand, for ‘color’ adjective metaphors that were predicted as having no change (0), the proportion of cases showing a change different from the predicted change was significantly higher than the proportion showing the predicted change, χ^2^ (1, N = 159) = 33.515, *p*<.001. Among the ‘color’ adjective metaphors that showed a change different from the predicted change, the proportion of cases showing a change toward the negative pole (−) was significantly higher than the proportion showing a change toward the positive pole (+), χ^2^ (1, N = 116) = 112.034, *p*<.001. This result suggests that only “color” adjective metaphors tend to change toward the negative pole against the prediction of no change (0).

**Table 4:** Comparison between predicted semantic changes and actual semantic changes for five types of adjective metaphors.

Types of adjective	Semantic intensity		Predicted change				Actual change			Sum
							0	+	−	
color	T = V		0				43	1	115	159
	T<V		+				144	39	53	236
	T>V		−				3	0	112	115
	Sum						190	40	280	510
touch	T = V	0					73	8	25	106
	T<V	+					56	65	3	124
	T>V	−					38	1	136	175
	Sum						167	74	164	405
sound	T = V			0			65	2	20	87
	T<V			+			44	52	0	96
	T>V			−			34	5	243	282
	Sum						143	59	263	465
taste	T = V				0		70	5	55	130
	T<V				+		75	64	15	154
	T>V				−		14	0	152	166
	Sum						159	69	222	450
smell	T = V					0	80	1	46	127
	T<V					+	47	10	5	62
	T>V					−	30	3	318	351
	Sum						157	14	369	540

Unit = cases of SD scale.

For the ‘color,’ ‘taste,’ and ‘smell’ adjective metaphors that were predicted to change toward the positive pole (+), the proportion of cases showing the predicted change was significantly lower than that showing a different change, χ^2^ (1, N = 236) = 105.779, *p*<.001 for color; χ^2^ (1, N = 154) = 4.389, *p*<.05 for taste; χ^2^ (1, N = 62) = 28.451, *p*<.001 for smell. As for ‘touch’ and ‘sound’ adjective metaphors, the proportion of cases showing the predicted change as the predicted change was slightly higher than that showing a different change, but this difference was not significant. This finding indicates that all of the adjective metaphor types do not show the predicted change toward the positive pole (+).

Regarding all of the metaphor types that were predicted to change toward the negative pole (−), the proportion of cases showing the predicted change was significantly higher than that showing a different change, χ^2^ (1, N = 115) = 103.313, *p*<.001 for color; χ^2^ (1, N = 175) = 53.765, *p*<.001 for touch; χ^2^ (1, N = 243) = 147.574, *p*<.001 for sound; χ^2^ (1, N = 166) = 114.722, *p*<.001 for taste; χ^2^ (1, N = 351) = 231.410, *p*<.001 for smell. This result confirms that actual semantic changes do in fact follow the predicted change toward the negative pole (−).

To see more clearly whether there was a tendency for adjective metaphors to evoke positive or negative effects, we classified all the cases showing different changes from the predicted change into those having a positive effect and those having a negative effect. Cases showing no change against the predicted change to the negative pole (−) were regarded as evoking a weakly positive effect, and were classified into the positive effect category in the same way as those which showed a change toward the positive pole (+) against the predicted change to the negative pole (−). Cases showing no change against the predicted change toward the positive pole (+) were regarded as evoking a weakly negative effect, and were classified into the negative effect category in the same way as those which showed a change toward the negative pole (−) against the predicted change to the positive pole (+). As a result, the 848 cases that showed changes different from the predicted changes were classified into 145 positive effect cases and 703 negative effect cases. A Chi-square test showed that the cases showing a negative effect were significantly more frequent than those showing a positive effect, χ^2^ (1, N = 848) = 367.175, *p*<.001. This result suggests that semantic interactions between the vehicles and the topics of the adjective metaphors tend to evoke a negative effect. In addition, we analyzed the tendency of negative effects among the adjective metaphor types. Table 5 shows the number of cases classified as showing either a positive or negative effect.

**Table 5:** Comparison among the five types of adjective metaphors showing unpredicted changes.

	Positive effect	Negative effect	Sum
color	4	312	316
touch	47	84	131
sound	41	64	105
taste	19	145	164
smell	34	98	132
Sum	145	703	848

Unit = cases of SD scales.

We conducted a Chi-square test using Bonferroni correction (alpha level of. 005) for the five types of adjective metaphors. The result showed that adjective metaphors created from adjectives denoting ‘color’ evoked the most negative effect. This result is consistent with the results of the analysis using the full dataset of 2370 cases. ‘Color’ adjective metaphors evoked a significantly more negative effect than the other four types of adjective metaphors, χ^2^ (1, N = 447) = 109.763, *p*<.001 for color vs. touch; χ^2^ (1, N = 421) = 117.848, *p*<.001 for color vs. sound; χ^2^ (1, N = 480) = 25.203, *p*<.001 for color vs. taste; χ^2^ (1, N = 448) = 71.947, *p*<.001 for color vs. smell. Differences among the color adjectives, *yellow* ‘kiiroi,’ *blue* ‘aoi,’ *red* ‘akai,’ *black* ‘kuroi,’ and *white* ‘shiroi,’ were not observed.

The second most negative effect was observed for adjective metaphors created from the adjectives denoting ‘taste’. These metaphors evoked significantly more negative effects than ‘touch,’ ‘smell,’ or ‘sound’; χ^2^ (1, N = 295) = 24.746, *p*<.001 for taste vs. touch; χ^2^ (1, N = 296) = 9.993, *p*<.005 for taste vs. smell; χ^2^ (1, N = 269) = 27.859, *p*<.001 for taste vs. sound. These negative effects were especially observed among adjective metaphors created from *sour* ‘suppai,’ *bitter* ‘shibui,’ and *hot* ‘karai.’

Significant differences among adjective metaphors created from the adjectives denoting ‘touch,’ ‘sound,’ and ‘smell’ were not observed; χ^2^ (1, N = 236) = .250, *p* = .617 for touch vs. sound; χ^2^ (1, N = 263) = 3.160, *p* = .075 for touch vs. smell; χ^2^ (1, N = 237) = 4.775, *p* = .029 for sound vs. smell. As for ‘touch,’ adjective metaphors showing a negative effect were observed more frequently among those created from *hard* ‘katai’ and *cold* ‘tsumetai’ than among those created from *light* ‘karui,’ *soft* ‘yawarakai,’ and *hot* ‘atsui.’ As for ‘smell,’ negative effects were observed among those created from *stinking (1)* ‘kinakusai,’ *stinking (2)* ‘kusai,’ and *smelly* ‘namagusai.’

The results of Experiment 1 showed that 848 cases did not show the expected shift toward the semantic value of the vehicle. One possible explanation of this unexpected change may be related to the imageability or frequency of the words. For example, metaphor ratings may shift towards the topic if the topic is more imageable than the vehicle. To examine whether such differences in imageability or frequency between topics and vehicle causes the unexpected semantic value shift, we conducted multiple regression analyses with semantic value shift as the dependent variable. One analysis was conducted for the cases showing the unexpected value shift. The imageability and frequency values are based on the NTT database: lexical properties of Japanese [38]. Table 6 shows the results of the regression analysis. The independent variables are differences in imageability or frequency between topics and vehicles. The result showed that the difference in imageability or frequency between topics and vehicles has no significant relation with the previously observed unexpected semantic value shift.

**Table 6:** Regression analysis of word imageability and frequency for unexpected semantic value shifts.

Variable	Unexpected value shift		
	*B*	*SE B*	*β*
1. Difference in imageability	−0.034	0.024	−0.047
2. Difference in frequency	−0.007	0.008	−0.029

*R^2^* = .003, *F* (2, 845) = 1.370, *P* = 0.254.

We also analyzed how the full dataset of 2,370 ratings are distributed amongst the 15 SD scales. The results are shown in Table 7. We conducted a Chi-square test using Bonferroni correction (alpha level of. 005) for the distribution of the semantic value shifts among the 15 SD scales. The scales ‘old – new,’ ‘ugly – beautiful,’ and ‘scary – safe’ tended to show the unexpected value shift, while ‘unclear – clear,’ ‘heavy – light,’ ‘dark – light,’ ‘not appropriate – appropriate,’ and ‘dull – sharp’ tended to frequently show the expected semantic value shift to a statistically significant degree. We speculate that this result suggests that sensuous, or emotion-oriented, SD scales tend to result more frequently in the unexpected value shift than property-oriented SD scales [37].

**Table 7:** Distribution of the unexpected and expected value shifts amongst the 15 SD scales.

15 SD scales	Unexpected value shift	Expected value shift	?^2^ value, N = 158
old - new	110	48	78.739***
ugly - beautiful	74	84	8.403**
scary - safe	69	89	4.280*
sad - glad	68	90	3.621
weak - powerful	60	98	0.331
coarse - delicate	58	100	0.059
dislike - like	54	104	0.176
not interesting - interesting	53	105	0.343
uncomfortable - comfortable	47	111	2.503
not salient - salient	45	113	3.663
unclear - clear	44	114	4.326*
heavy - light	43	115	5.044*
dark - light	42	116	5.817*
not appropriate - appropriate	41	117	6.645**
dull - sharp	40	118	7.529**

*P<.05, **P<.01, ***P<.001.

We must acknowledge the possibility that the meaning of some of the scales may have been unclear to the participants, and thus we do not know with certainty if everyone interpreted the scales in the same way. The results of Experiment 1, however, showed that semantic interactions between vehicles denoting the different perceptual domains and adjective metaphor topics tended to evoke negative cognitive effects, and that there were differences among the types of adjective metaphors. This raises the question of why and through what processes such cognitive effects are evoked. One possible explanation for these observed differences among the types of adjective metaphors is different degrees of accessibility among the adjective metaphor types. According to Ullmann’s [24] theory of directionality, a metaphor with a source domain lower in the hierarchy of sense modalities than the target domain should tend to be cognitively more accessible than a metaphor with the reverse direction of domains. Figure 1 shows this directionality, as proposed by Williams [25].

Our finding that the ‘color’ adjective metaphors evoked the most negative effect may be related to the fact that color is located in the highest position of the hierarchy. However, the second most negative effect was evoked by adjective metaphors created from ‘taste,’ which is located in a lower position in the hierarchy. Furthermore, as we described in Methods section, in our pre-experiment we asked participants to evaluate how easily they understood the metaphors proposed for the experiment materials as well as how conventional they felt the proposed metaphors were, and selected metaphors with a mean value ranging from −2.0 to +2.0 in both scales. Through this procedure, we reduced the possibility that differences in the cognitive effects evoked by the adjective metaphors would result from differences in the accessibility or conventionality among the materials used in the experiment. Therefore, the observed different effects evoked by the five types of adjective metaphors cannot be explained simply by differences in acceptability among the adjective metaphor types. This observation is supported by the result of the multiple regression analysis using imageability and frequency described above.

As stated above, the most negative effect was evoked by the adjective metaphors created from ‘color.’ This result is interesting because, according to Sakamoto [2] and Wierzbicka [39], color adjectives such as *yellow* ‘kiiroi,’ *blue* ‘aoi,’ *red* ‘akai,’ and *white* ‘shiroi’ do not themselves have explicit negative meanings. Wierzbicka [39] states, for example, that the meaning of ‘yellow’ is based on our experience of things that are yellow, such as the sun, and that the meaning of ‘blue’ is similarly based on our experiences of things that are blue, like the sky.

The results of our experiment are not particularly unusual; in fact, they are consistent with those of Sakamoto [2]. Sakamoto [2] analyzed the meanings of composite expressions of nouns modified by color terms (e.g., red, blue, yellow, black, and white) collected from a Japanese corpus containing literary texts. She found that a number of Japanese color metaphors have meanings that are not predictable from those typically associated with color terms, as pointed out by Wierzbicka [39]. This finding suggests that color terms tend to modify nouns with negative images and that color metaphors therefore tend to emphasize negative images. To verify the results of this corpus-based analysis, she subsequently conducted psychological experiments using Japanese color metaphors composed of nouns with neutral images. Japanese respondents were asked to name images associated with the color metaphors, and their answers were compared with images evoked by color terms. Results showed that color metaphors were associated with negative and different images from those that the color terms were associated with.

Furthermore, as mentioned in the Introduction, Trakl has used color adjectives to emphasize negative concepts such as death and grief throughout his poems [1], [40]. These German examples suggest that such cognitive effects evoked by adjective metaphors created from color metaphors are not peculiar to Japanese and therefore could be universal. Acknowledging that the symbolic meanings of each color are different across cultures, namely, from language to language, Wescott [41] points out that there are some universal color metaphors and that most of them are more negative than positive in nature. The same color can have quite different metaphorical meanings in different languages, and the same meaning can have quite different color representations in different languages. Nonetheless, according to Wescott [41], uninhibited anger makes the skin red from the outward rush of blood (as exemplified by English, Italian, and Yoruba metaphors). Inhibited anger makes the skin either white from muscular constriction (as exemplified by a German metaphor) or blue from shallow and under-oxygenated breathing (as exemplified by a Latin metaphor). Anger that causes the angered individual to feel sick gives the skin a green or yellow tone (as exemplified by a German metaphor), and scowling in anger causes parts of the face to be in shadow and thus makes the facial skin appear black (as exemplified by another German metaphor). In fact, a number of colors are widely used metaphorically to indicate anger. The results of our research, therefore, suggest that the cognitive effects of adjective metaphors are worth exploring across languages.

In Experiment 1 we analyzed how semantic interactions between the vehicles and topics of adjective metaphors function to shift the meanings of words to either the positive pole or the negative pole. The results showed that adjective metaphors, and especially those modified by color adjectives, tend to evoke negative images, thereby contradicting our prediction. In Experiment 2, therefore, we examined whether evoking negative meanings is a unique feature of adjective metaphors through a comparison with nominal and predicative metaphors.

### Experiment 2

Because our aim was to examine whether the evoking of negative meanings is a unique feature of adjective metaphors, we selected the SD scales shown in Table 8, which were clear and appropriate for measuring whether metaphorical expressions evoke negative meanings. Details of the selection process are given in File S1.

**Table 8:** Seven SD scales used in the experiment.

dark - light	dislike- like	inelegant – elegant
sad - glad	ugly - beautiful	uncomfortable - comfortable
bad - good		

We also selected topics and vehicles to construct Japanese metaphorical expressions. The topics were selected from nouns categorized as belonging to the highly abstract semantic level in a Japanese thesaurus [42]. From these nouns, we selected additional nouns of high familiarity [38]. In this experiment, we wanted to examine how the semantic interactions between the topics and vehicles of three types of metaphors function to shift the meanings of the nouns as topics to either the positive or negative semantic pole. We thus selected the following four nouns with neutral meanings to be used as topics: *smell* (‘nioi’), *(the current)moment* (‘genzai’), *footstep* (‘ashioto’), and *pose* (‘shisei’). Details of the process in which nouns with neutral meanings were selected are given in File S2.

Vehicles for the three types of metaphors were also selected from the Japanese thesaurus [42]. We selected 19 adjectives, 15 nouns, and 15 verbs. The selected 49 vehicles are listed in File S3.

Next we constructed metaphors by combining the four topics and the 49 vehicles. We excluded 16 expressions that were not regarded as metaphors, such as “minor smell” and “moment of life.”

Using the same procedure as Experiment 1, participants were asked to evaluate how easily they understood each metaphor. The ratings were made on a seven-point scale ranging from −3 (very difficult) through 0 (not sure) to +3 (very easy). They were also asked to evaluate how conventional they felt each metaphor to be. The ratings were made on a seven-point scale from −3 (not at all conventional) through 0 (not sure) to +3 (highly conventional). As a result, we confirmed that all the metaphors were rated from −2.0 to +2.0 in the accessibility and conventionality scales. This procedure reduced the possibility that differences in the cognitive effects evoked by metaphors would result from differences in the accessibility or conventionality among the materials used in the experiment.

We conducted a psychological experiment in which participants evaluated the meaning of all three types of metaphors (e.g., nominal metaphors: *smell of dreams* (‘yume no nioi’), predicative metaphors: *rolling smell* (‘korogaru nioi’), adjective metaphors: *white smell* (‘shiroi nioi’)). These metaphors were constructed by combining the 4 topics and the words associated with the 3 types of vehicles, namely, nouns, verbs, and adjectives. In the psychological experiment, participants were asked to evaluate the meaning of the metaphorical expressions. A total of 60 Japanese males and females, aged 20–28, were classified into two groups, and 90 metaphorical expressions were assigned to each group. Participants were asked to rate the assigned expressions against 7 SD scales given in Table 7. The ratings were made on a seven-point scale ranging from −3 through 0 to +3. We regarded the value −3 as the negative semantic pole and the value +3 as the positive semantic pole.

We also conducted another experiment to specify the meanings of the individual vehicles in isolation. In the experiment, 30 Japanese males and females, aged 20–24, were asked to rate the 49 vehicles against the seven SD scales. The ratings were made on a seven-point scale ranging from −3 through 0 to +3. We regarded the value −3 as the negative semantic pole and the value +3 as the positive semantic pole.

Based on the results of the experiments, we analyzed whether the semantic interactions between the vehicles and topics cause the neutral meanings of topics to change to a negative or positive meaning.

We regarded the mean values in the seven SD scales given in Table 8 as indicating the evaluation values of the metaphorical expressions and vehicles. We classified the metaphorical expressions into cases showing no semantic change, those showing a change toward the positive semantic pole, and those showing a change toward the negative semantic pole. We conducted a t-test (two-tailed, alpha level of. 05) to investigate semantic changes evoked by the semantic interaction between the topics and vehicles. Because only the topics with neutral meanings were selected during the pre-experiment, metaphorical expressions that had no significant difference between their mean value and value 0 were regarded as metaphors showing no semantic change (0). In addition, metaphorical expressions that had a significant difference between their mean values and value 0 were classified into either metaphors showing a change toward the positive semantic pole or those showing a change toward the negative semantic pole.

We also classified vehicles into those with neutral meanings, those with positive meanings, and those with negative meanings. Using t-tests (two-tailed, alpha level of. 05), vehicles that had no significant difference between their mean value and value 0 were regarded as vehicles with a neutral meaning (0). In addition, vehicles that had a significant difference between their mean value and value 0 were classified into either vehicles with a positive meaning or those with a negative meaning.

We assume that the meanings of metaphors result from the semantic interaction between their vehicles and topics. Because the meanings of the individual topics used in our study were neutral, we classified all of the metaphorical expressions into those using vehicles with neutral meanings, those using vehicles with positive meanings, and those using vehicles with negative meanings. Table 8 shows the results of this classification.

As for the metaphors in which the vehicles by themselves had neutral meanings, as shown in Table 9, the proportion of the metaphors showing a neutral meaning was the highest. A Chi-square test was conducted among the expressions showing +, −, and neutral (0) meanings. Results showed that metaphorical expressions with neutral meanings (0) were observed significantly more frequently than metaphorical expressions with positive meanings (+), χ^2^ = (1, N = 49) = 37.735, p<.01 (0 vs. +). However, there was no significant difference found between the number of neutral metaphorical expressions (0) and the number of negative (−) ones, χ^2^ = (1, N = 78) = 2.513, p>.05 (0 vs. −).

**Table 9:** Number of metaphors showing neutral, positive, and negative meanings.

Meaning of vehicles		+	−	0	Sum
Neutral	Nominal metaphors	1	7	18	26
	Predicative metaphors	1	8	18	27
	Adjective metaphors	1	17	10	28
	Sum	3	32	46	81
Positive	Nominal metaphors	25	1	8	34
	Predicative metaphors	8	1	5	14
	Adjective metaphors	8	3	6	17
	Sum	41	5	19	65
Negative	Nominal metaphors	0	0	0	0
	Predicative metaphors	1	14	4	19
	Adjective metaphors	0	12	3	15
	Sum	1	26	7	34

As for nominal metaphors, the proportion of metaphors showing a neutral meaning was highest. The results of Chi-square tests showed that these metaphorical expressions (0) were significantly more common than the other expressions, χ^2^ = (1, N = 19) = 15.211, p<.01 (0 vs. +); χ^2^ = (1, N = 25) = 4.840, p<.05 (0 vs. −).

As for predicative metaphors, the proportion of metaphors showing a neutral meaning was highest. The results of Chi-square tests showed that these metaphorical expressions (0) were significantly more common than the positive (+) metaphorical expressions, χ^2^ = (1, N = 19) = 0.154, p<.01 (0 vs. +); moreover, there was a slightly significant difference between the number of neutral (0) metaphorical expressions and the number of negative (−) ones, χ^2^ = (1, N = 26) = 3.846, p = .05 (0 vs. −).

As for adjective metaphors, the proportion of metaphors showing a negative meaning was highest. The results of Chi-square tests showed that these metaphorical expressions (−) were significantly more common than the positive (+) metaphorical expressions, χ^2^ = (1, N = 18) = 14.222, p<.01 (− vs. +). However, there was no significant difference found between the number of neutral expressions (0) and the number of negative (−) ones, χ^2^ = (1, N = 27) = 1.815, p>.05 (0 vs. −).

These results show that nominal metaphors and predicative metaphors are both affected by the meaning of their vehicles and tend to show neutral meanings, while adjective metaphors tend to show negative meanings.

Chi-square tests were conducted among the three types of metaphors. The results showed that adjective metaphors showed significantly more frequent negative meanings than the other two types of metaphors (χ^2^ = (1, N = 54) = 6.234, p<.05 for adjective metaphors vs. nominal metaphors; χ^2^ = (1, N = 55) = 5.357, p<.05 for adjective metaphors vs. predicative metaphors).

The analyses of metaphors using vehicles with neutral meanings showed that nominal metaphors and predicative metaphors both tend to show neutral meanings, while adjective metaphors tend to show negative meanings. The tendency of adjective metaphors to show negative meanings was clearly revealed by the Chi-square tests among the three types of metaphors. Therefore, the results of our analyses suggest that, unlike predicative and nominal metaphors, adjective metaphors tend to evoke negative meanings even when their individual vehicles have neutral meanings.

As for metaphors with vehicles having positive meanings (See Table 9), the proportion of the metaphors showing a positive meaning was the highest. Chi-square tests were conducted among the expressions showing +, −, and neutral (0) meanings. Results showed that metaphorical expressions showing positive meanings (+) were observed significantly more frequently than the other metaphorical expressions, χ^2^ = (1, N = 46) = 28.174, p<.01 (+ vs. −); χ^2^ = (1, N = 60) = 8.067, p<.01 (+ vs. 0).

As for nominal metaphors, the proportion of metaphors showing a positive meaning was highest. The results of Chi-square tests showed that these positive metaphorical expressions (+) were significantly more common than the other expressions, χ^2^ = (1, N = 26) = 22.154, p<.01 (+ vs. −); χ^2^ = (1, N = 33) = 8.758, p<.01 (+ vs. 0).

As for predicative metaphors, the proportion of metaphors showing a positive meaning was highest. However, there was no significant difference found among the other types of metaphors, χ^2^ = (1, N = 14) = 5.286, p>.05.

As for adjective metaphors, the proportion of metaphors showing a positive meaning was highest. However, there was no significant difference found among the other metaphors, χ^2^ = (1, N = 17) = 2.235, p>.05.

Although the proportion of nominal metaphors showing positive meanings was found to be the highest, Chi-square tests showed that there was no significant difference among the nominal metaphors and other metaphors (χ^2^ = (1, N = 58) = 1.239, p>.05 for nominal metaphors vs. predicative metaphors; χ^2^ = (1, N = 51) = 3.477, p>.05 for nominal metaphors vs. adjective metaphors).

These results show that for vehicles with positive meanings, all three types of metaphors tend to show positive meanings. This result suggests that metaphors using vehicles with positive meanings tend to be affected by the meaning of their vehicles.

As for metaphors with vehicles having negative meanings (See Table 9), the proportion of the metaphors showing a negative meaning was the highest. Chi-square tests were conducted among the expressions showing +, −, and neutral (0) meanings. Results showed that metaphorical expressions showing negative meanings (−) were observed significantly more frequently than the other metaphorical expressions, χ^2^ = (1, N = 27) = 23.148, p<.01 (− vs. +); χ^2^ = (1, N = 33) = 10.939, p<.01 (− vs. 0).

As for nominal metaphors, we were unable to find metaphors using vehicles that were rated negative by the participants of the experiment.

As for predicative metaphors, the proportion of metaphors showing a negative meaning was highest. Chi-square tests showed that negative metaphorical expressions (−) were significantly more common than the other expressions, χ^2^ = (1, N = 15) = 11.267, p<.01 (− vs. +); χ^2^ = (1, N = 18) = 5.556, p<.05 (− vs. 0).

As for adjective metaphors, the proportion of metaphors showing a negative meaning was highest. Chi-square tests showed that negative metaphorical expressions (−) were significantly more common than the other expressions, χ^2^ = (1, N = 15) = 5.400, p<.05 (− vs. 0).

We further classified all the metaphors into either metaphors showing negative meanings or metaphors having positive and neutral meanings, and then compared the predicative metaphors and adjective metaphors using a Chi-square test.

The results of the Chi-square test showed no significant difference between predicative metaphors and adjective metaphors, χ^2^ = (1, N = 34) = .186, p>.05.

This result suggests that metaphors using vehicles with negative meanings tended to be affected by the meaning of their vehicles. This tendency is the same for both nominal and predicative metaphors using vehicles with neutral meanings as well as for all of the metaphors using vehicles with positive meanings.

In Experiment 2 we analyzed whether the evoking of negative meanings is a unique feature of adjective metaphors through a comparison with nominal and predicative metaphors. The results showed that the meanings of metaphors are generally affected by the meanings of their vehicles, and that all types of metaphors using vehicles with positive meanings tend to evoke positive meaning while all types of metaphors using vehicles with negative meanings tend to evoke negative meanings. However, as for metaphors with vehicles of neutral meaning, adjective metaphors evoked negative meanings significantly more frequently than both nominal and predicative metaphors. The results of Experiment 2 were thus consistent with those of Experiment 1, thereby verifying our hypothesis that the evoking of negative meanings is a unique feature of adjective metaphors. However, it is still unclear why negative meanings are evoked by adjective metaphors. Because adjective metaphors evoking the most negative meanings are used in very striking ways, such as in Trakl’s poems as well as Japanese literary texts introduced in the Introduction, we hypothesized that negative meanings evoked by adjective metaphors are related to poeticness. We tested this hypothesis in Experiment 3.

### Experiment 3

The results of Experiment 2 suggested that the meanings of metaphors are generally affected by the meanings of their vehicles, and previous studies such as Osgood [35] and Becker [34] have also found that the meanings of metaphors are affected by the meanings of their vehicles. Therefore, in Experiment 3 we focused on the characteristics of metaphors that evoke negative meanings despite being constructed from vehicles with originally positive meanings.

We selected 15 vehicles that were evaluated as significantly positive in a pre-experiment (File S4). Similarly, we also selected nouns with neutral meanings to be used as seven topics through a pre-experiment (File S5).

We combined the vehicles and topics selected through the pre-experiments. Among 105 expressions, five expressions regarded as non-metaphors were excluded and we selected 100 adjective metaphors, including the following: *red silence* (“akai seijaku”), *blue taste* (“aoi aji”), *white today* (“shiroi genzai”), *beautiful footstep* (“utsukushii ashioto”), *bright touch* (“akarui shokkaku”), *soft intention* (“yawarakai honne”), *warm thinking* (“atatakai kangae”), *sweet silence* (“amai seijaku”), *delicious intention* (“oishii honne”), *crispy footstep* (“koobashii ashioto”), *safe thinking* (“anzenna kangae”), *gentle today* (“yasashii genzai”), *quiet taste* (“shizukana aji”), *pleasant footstep* (“tanoshii ashioto”), and *new silence* (“atarashii seijaku”).

Using the same procedure as for Experiments 1 and 2, participants were asked to evaluate how easily they understood each metaphor. The ratings were made on a seven-point scale ranging from −3 (very difficult) through 0 (not sure) to +3 (very easy). They were also asked to evaluate how conventional they felt each metaphor to be. The ratings were made on a seven-point scale from −3 (not at all conventional) through 0 (not sure) to +3 (highly conventional). We confirmed all the metaphors were from −2.0 to +2.0 on the accessibility scale and the conventionality scale and we ensured that the metaphors used in the experiments were neither incomprehensible nor too conventional.

In Experiment 3 we tested the hypothesis that negative meanings evoked by adjective metaphors are related to poeticness. First we conducted a psychological experiment to grasp the participants’ basic impressions of the adjective metaphors. In the experiment, 60 Japanese males and females, aged 18–25, were asked to rate the meanings of the 100 adjective metaphors on a seven-point scale ranging from −3 (extremely negative) through 0 to +3 (extremely positive).

Next, we conducted another psychological experiment to assess the poeticness of the 100 adjective metaphors. Fifty Japanese males and females, aged 20–24, participated in the experiment. These participants were different from those who participated in the previous impression rating experiment described above. They were asked to rate how poetic they felt each metaphor was on a seven-point scale ranging from −3 (not at all poetic) through 0 (not sure) to +3 (highly poetic).

We conducted a correlation analysis between the poeticness ratings and the meaning evaluations of the adjective metaphors. Our hypothesis that negative meanings evoked by adjective metaphors are related to poeticness predicted that adjective metaphors would be rated as more poetic, and thereby evaluated as more negative, that is, closer to the negative pole on the impression scale. Thus, our hypothesis predicted that there would be a negative correlation between the evaluations of poeticness and the meanings of the adjective metaphors. The results showed that the correlation coefficient for poeticness and a negative evaluation was *r* = −0.336 (*p*<.05), and that the more poetic metaphors were evaluated, the more negatively they were evaluated.

The results of Experiment 3 suggested the possibility that negative meanings evoked by adjective metaphors are related to poeticness, and that poetic metaphors evoke negative meanings more easily than less poetic metaphors. However, we do not know with certainty whether participants who felt that a specific metaphor is poetic tended to rate that metaphor negatively, because the ratings were collected from different groups of participants.

The results of this study seem to be related to the results of Utsumi et al. [43]. Utsumi et al. [43] focused on two types of metaphors–explanatory metaphors and literary or figurative metaphors–that accomplish different discourse goals. They demonstrated that the production of literary metaphors requires the activation of both prototypical and less prototypical members of the category characterized by the topic property, while the production of explanatory metaphors does not. They claim that this processing difference provides one explanation for their finding that explanatory metaphors generated in a metaphor production experiment were more prototypical than literary metaphors. In other words, less prototypical members of the category are activated during the processing of literary metaphors, and as a result less prototypical meanings of vehicles are selected. Because the poetic metaphors used in our study can be considered literary metaphors, the results of Utsumi et al. [43] lead us to further suppose that less prototypical meanings of vehicles are activated during the processing of poetic metaphors. Because the vehicles used in Experiment 3 were all evaluated as significantly positive, the prototypical meanings of their vehicles are also assumed to be positive. Therefore, it seems natural that negative meanings, i.e., less prototypical vehicle meanings, would be activated during the processing of poetic adjective metaphors.

## Results and Discussion

While numerous other cognitive studies of metaphors have focused on nominal metaphors such as “*My job is a jail*” [13]–[15], [17] or predicative metaphors such as “He *shot down* all of my arguments” [3], [18], [19], our research is one of the few studies to focus on the cognitive effects evoked by adjective metaphors; most studies examining adjective metaphors since Ullmann [24] and Williams [25] have focused on how their acceptability as synesthetic metaphors can be explained by the pairing of the modalities of the adjective modifiers and head nouns. Through Experiments 1 and 2, we showed that adjective metaphors evoke negative meanings and, with respect to metaphors whose vehicles have neutral meanings themselves, that adjective metaphors evoked negative meanings significantly more frequently than both nominal metaphors and predicative metaphors. However, here we must also acknowledge the possibility that the meaning of some of the scales may have been obscure, and thus we do not know with certainty if everyone interpreted the scales in the same way. As for Experiment 1, there is one other limitation that should be mentioned; because the stimuli were rated by different groups of participants, the collected ratings were similarly performed by those different groups.

In Experiment 3, we explored the reason why adjective metaphors evoked negative meanings. The results suggested the possibility that negative meanings evoked by adjective metaphors are related to poeticness, and that poetic metaphors evoke negative meanings more easily than less poetic metaphors. However, we do not know with certainty whether participants who felt that a specific metaphor is poetic tended to rate that metaphor negatively, because the ratings were collected from different groups of participants. This result may be related to the results found by Utsumi et al. [43]; in that study, explanatory metaphors are used to clarify certain properties of the topic, while literary metaphors, namely poetic metaphors, are used to evoke an aesthetically pleasing feeling by enriching the meanings conveyed by the metaphors. Their psychological experiments demonstrated that the production of literary metaphors required activation of both prototypical and less prototypical members of the category characterized by the topic property, while the production of explanatory metaphors did not. If adjective metaphors were apt to be used for poetic and literary purposes, their finding that less prototypical members of the category are activated during the process of poetic and literary metaphors might be a clue to solving our question of why adjective metaphors evoke negative meanings. In addition, in our study the adjective metaphors found to evoke the most negative effect were those created from colors. According to Sakamoto [2] and Wierzbicka [39], color adjectives such as yellow ‘kiiroi,’ blue ‘aoi,’ red ‘akai,’ and white ‘shiroi’ do not themselves have explicit negative meanings, and the prototypical meanings of color adjectives are rather positive. Wierzbicka [39] states that the meaning of ‘yellow’ is based on our experience of things that are yellow, such as the sun, and that the meaning of ‘blue’ is similarly based on our experiences of things that are blue, like the sky. In other words, the negative meanings of color adjectives are less prototypical. The color metaphors we discussed in the Introduction were literary metaphors used in poems by Trakl, and they were used to emphasize and enrich negative meanings such as death and grief. The goal of the adjective metaphors used by Trakl, namely the goal of evoking an aesthetically pleasing feeling by enriching the meanings conveyed by the literary metaphors, is only achieved when readers search for less prototypical meanings of the colors. If the prototypical meanings of colors such as those used to describe the sun or the sky directly affected the meanings of color metaphors, then readers should search for less prototypical meanings of colors, namely negative meanings, when they find the metaphors to be poetic and literary in the processing of their meanings.

The mechanisms of the negative effects evoked by adjective metaphors can be discussed in relation to the comprehension process revealed by previous research. A considerable number of cognitive studies on metaphors have examined the cognitive mechanism of metaphor comprehension. Most of these studies have focused on the comprehension process of nominal and predicative metaphors. Glucksberg and his colleagues [14], [44] argue that individuals comprehend nominal metaphors via a categorization process. In this categorization process, individuals understand nominal metaphors by seeing the target concept as belonging to the superordinate metaphorical category exemplified by the source concept. As for the mechanisms of adjective metaphors, Utsumi and Sakamoto [18], [45], [46] argue that they can be explained as a two-stage categorization process. In the case of “*red voice*” created from the neutral vehicle “red,” for example, the adjective “*red*” first evokes an intermediate category “*red things,*” to which “*blood,*” “*fire,*” “*passion,*” “*apple,*” and “*danger,*” typically belong. Then, exemplars relevant to the noun “*voice*” are selected, and these then evoke a final abstract category of property, such as “*scary,*” “*screaming,*” and “*dangerous.*” In this way, adjective metaphors are understood as not being directly mapped onto their topics from ad hoc categories of vehicles, but rather by mediating to an intermediate category. Therefore, supposing that adjective metaphors are comprehended in this two-stage categorization process, it seems reasonable that the prototypical meanings of their vehicles do not directly affect the meanings of adjective metaphors, and that adjective metaphors are more likely to evoke different meanings compared with the meanings of their vehicles by themselves, namely, the exemplars with negative meanings are selected among the various exemplars belonging to the intermediate category evoked by the adjectives as vehicles.

## Conclusions

This paper explored the cognitive effects evoked by adjective metaphors. Through a series of psychological experiments, we showed that adjective metaphors evoke negative meanings and, with respect to the metaphors for which their vehicles themselves have a neutral meaning, adjective metaphors evoked negative meanings significantly more frequently than both nominal and predicative metaphors. Our research is one of only a few studies to examine adjective metaphors, while many cognitive studies on metaphors have focused on nominal and predicative metaphors. Our research is also one of only a few studies to focus on the cognitive effects evoked by adjective metaphors; most other studies examining adjective metaphors have focused on how the acceptability of adjective metaphors as synesthetic metaphors can be explained by the pairing of the modalities of the adjective modifiers and head nouns. The results of our study have implications for future studies of metaphors, which represent one of the most basic human cognitive abilities.

## Supporting Information

File S1 Details of the SD scales used in Experiment 2.
(DOCX)

File S2 Details of the nouns with neutral meanings used in Experiment 2.
(DOCX)

File S3 Vehicles used in Experiment 2.
(DOCX)

File S4 Vehicles with positive meanings used in Experiment 3.
(DOCX)

File S5 Topics with neutral meanings used in Experiment 3.
(DOCX)
