# Supplementary material for: Adjective Metaphors Evoke Negative Meanings
Source: PLoS One. 2014 Feb 19;9(2):e89008. doi: 10.1371/journal.pone.0089008 (PMC3929652; doi:10.1371/journal.pone.0089008)
Supplement: File S1 — Details of the SD scales used in Experiment 2. (DOCX) [file pone.0089008.s001.docx]

Supporting Information S1: Details of the SD scales used in Experiment 2

We conducted a pre-experiment using the following 32 SD scales, which were selected from Kusumi [36] using the same procedure as Experiment 2, but excluding scales such as “blue – yellow”.

SD scales: “dislike – like,” “uncomfortable – comfortable,” “ugly – beautiful,” “dark – light,” “sad – glad,” “bad – good,” inelegant – elegant,” “not interesting – interesting,” “unclear – clear,” “not appropriate – appropriate,” “dull – sharp,” “persistent – tidy,” “low – high,” coarse – delicate,” “not bright – bright,” “unnecessary – necessary,” “small – big,” “heavy – light,” “not salient – salient,” “moist – dry,” “shallow – deep,” “temporary – eternal,” “big – little,” “square – circular,” “demonstrative – cover,” “not mysterious – mysterious,” “motionless – moving,” “relieved – anxious,” “fearful – benign,” “low – high,” “free – constrained,” and “new – old.” Ten Japanese males and females, aged 21–35 years, participated in the pre-experiment. Participants were asked to choose the SD scales for which they could easily identify a semantic pole as either positive or negative. Based on their responses, we decided to use the seven SD scales shown in Table 8, which were chosen by at least nine of the participants.
