# Supplementary material for: Adjective Metaphors Evoke Negative Meanings
Source: PLoS One. 2014 Feb 19;9(2):e89008. doi: 10.1371/journal.pone.0089008 (PMC3929652; doi:10.1371/journal.pone.0089008)
Supplement: File S2 — Details of the nouns with neutral meanings used in Experiment 2. (DOCX) [file pone.0089008.s002.docx]

Supporting Information S2: Details of the nouns with neutral meanings used in Experiment 2

We conducted an experiment to identify nouns with neutral meanings. Participants in this pre-experiment rated the meanings of the 54 nouns, which included *gloss* (‘tuya’), *taste* (‘aji’), *noise* (‘oto), *voice* (‘koe’), *touch* (‘tezawari’), and *heart* (‘kokoro’). In the pre-experiment, 15 Japanese males and females, aged 20 – 24, were asked to rate the 54 words against the seven SD scales. The ratings were made on a seven-point scale ranging from −3 through 0 to +3. We regarded the value −3 as the negative semantic pole and the value +3 as the positive semantic pole. Then, we conducted t-tests (two-tailed, alpha level of .05) and regarded the words having no significant difference between the mean semantic values of the words and “0” as topics with neutral meanings. As a result of this, *smell* (‘nioi’), *moment* (‘genzai’), *footstep* (‘ashioto’), and *pose* (‘shisei’) were selected.
