# Supplementary material for: Adjective Metaphors Evoke Negative Meanings
Source: PLoS One. 2014 Feb 19;9(2):e89008. doi: 10.1371/journal.pone.0089008 (PMC3929652; doi:10.1371/journal.pone.0089008)
Supplement: File S3 — Vehicles used in Experiment 2. (DOCX) [file pone.0089008.s003.docx]

Supporting Information S3: Vehicles used in Experiment 2

Adjectives: *shallow* (‘asai’), *light* (‘karui’), *far* (‘tooi’), *deep* (‘hukai’), *much* (‘ooi’), *circular* (‘marui’), *big* (‘ookii’), *small* (‘chiisai’), *heavy* (‘omoi’), *long* (‘nagai’), *short* (‘mijikai’), *white* (‘shiroi’), *red* (‘akai’), *black* (‘kuroi’), *blue* (‘aoi’), *large* (‘hiroi’), *narrow* (‘semai’), *near* (‘chikai’), and *minor* (‘sukunai’).

Nouns: *life*(1) (‘jinsei’), *music* (‘ongaku’), *world* (‘sekai’), *life*(2) (‘inochi’), *adventure* (‘bouken’), *destiny* (‘unmei’), *legend* (‘densetu’), *dream* (‘yume’), *joke* (‘joudan’), *literature* (‘bungaku’), *philosophy* (‘tetugaku’), *rusticity* (‘soboku’), *heart* (‘kokoro’), *image* (‘ime-ji’), and *intuition* (‘chokkan’).

Verbs: *float* (‘ukabu’), *flow* (‘nagareru’), *drift* (‘tadayou’), *circle* (‘mawaru’), *roll* (‘korogaru’), *tower* (‘sobieru’), *quake* (‘hurueru’), *swell* (‘takamaru’), *flip* (‘hikkurikaeru’), *fly around* (‘tobimawaru’), *break away* (‘kakedasu’), *shake* (‘yureru’), *swirl* (‘uzumaku’), *run* (‘furisosogu’), and *wave* (‘namiutu’).
