# Supplementary material for: Adjective Metaphors Evoke Negative Meanings
Source: PLoS One. 2014 Feb 19;9(2):e89008. doi: 10.1371/journal.pone.0089008 (PMC3929652; doi:10.1371/journal.pone.0089008)
Supplement: File S4 — Vehicles with positive meanings used in Experiment 3. (DOCX) [file pone.0089008.s004.docx]

Supporting Information S4: Vehicles with positive meanings used in Experiment 3

The vehicle candidates were 36 Japanese adjectives denoting perceptions spanning the five sense modalities selected from a Japanese thesaurus [1]. To clarify the meanings of the vehicles we conducted a psychological experiment. In the experiment, 10 Japanese males and females, aged 22–24, were asked to rate the meanings of the vehicles on a seven-point scale ranging from −3 (extremely negative) through 0 (not sure) to +3 (extremely positive). We conducted t-tests (two-tailed, alpha level of .05) between their mean values and “0” and selected those vehicles evaluated as significantly positive. The following 15 adjectives were selected: *red* (“akai”), *blue* (“aoi”), *white* (“shiroi”), *beautiful* (“utsukushii”), *bright* (“akarui”), *soft* (“yawarakai”), *warm* (“atatakai”), *sweet* (“amai”), *delicious* (“oishii”), *crispy* (“koobashii”), *safe* (“anshinna”), *gentle* (“yasashii”), *quiet* (“shizukana”), *pleasant* (“tanoshii”) and *new* (“atarashii”).

[1] Yamaguchi T (2003) Nihongo Dai-Thesaurus (Japanese Thesaurus). Tokyo: Taishukan Shoten.
