# Supplementary material for: Adjective Metaphors Evoke Negative Meanings
Source: PLoS One. 2014 Feb 19;9(2):e89008. doi: 10.1371/journal.pone.0089008 (PMC3929652; doi:10.1371/journal.pone.0089008)
Supplement: File S5 — Topics with neutral meanings used in Experiment 3. (DOCX) [file pone.0089008.s005.docx]

Supporting Information S5: Topics with neutral meanings used in Experiment 3

The topic candidates were 49 Japanese abstract nouns selected from the same Japanese thesaurus [1] that were known to have high familiarity [2]. We conducted another pre-experiment to find nouns with neutral meanings. Participants in the pre-experiment rated the meanings of the 49 nouns. Ten Japanese males and females, aged 21–37, were asked to rate the meanings of the 49 nouns on a seven-point scale ranging from −3 (extremely negative) through 0 (not sure) to +3 (extremely positive). We conducted t-tests (two-tailed, alpha level of .05) between their mean values and “0” and selected the nouns that had no significant difference from “0” when compared with those with neutral meanings. The following seven nouns were selected as topics with neutral meanings to be used in Experiment 3: *silence* (“seijaku”), *taste* (“aji”), *today* (“genzai”), *footstep* (“ashioto”), *touch* (“shokkaku”), *intention* (“honne”) and *thinking* (“kangae”).

[1] Yamaguchi T (2003) Nihongo Dai-Thesaurus (Japanese Thesaurus). Tokyo: Taishukan Shoten.

[2] Amano S, Kondo T (1999) NTT database series Nihongo-no Goi-Tokusei (Lexical properties of Japanese) Vol.1 Tokyo: Sanseido.
